# A Turn-ON fluorometric biosensor based on ssDNA immobilized with a metal phenolic nanomaterial for the sequential detection of Pb(ii) and epirubicin cancer drug†

**DOI:** 10.1039/d1ra00939g

**Published:** 2021-03-29

**Authors:** A. Arunjegan, P. Rajaji, S. Sivanesan, P. Panneerselvam

**Affiliations:** Department of Chemistry, SRM Institute of Science and Technology Kattankulathur Tamil Nadu 603 203 India panneerp1@srmist.edu.in panneerchem82@gmail.com +91 9688538842; Department of Applied Science and Technology, A. C Technology, Anna University Chennai Tamil Nadu 600 025 India

## Abstract

In this paper, we propose a fluorescent biosensor for the sequential detection of Pb^2+^ ions and the cancer drug epirubicin (Epn) using the interactions between label-free guanine-rich ssDNA (LFGr-ssDNA), acridine orange (AO), and a metal–phenolic nanomaterial (*i.e.*, nano-monoclinic copper–tannic acid (NMc-CuTA)). An exploration of the sensing mechanism shows that LFGr-ssDNA and AO strongly adsorb on NMc-CuTA through π–π stacking and electrostatic interactions, and this results in the fluorescence quenching of AO. In order to sense the target Pb^2+^, initially, LFGr-ssDNA specifically binds with Pb^2+^ ions to form a G4 complex (G–Pb^2+^–G base pair), which was released from the surface of NMc-CuTA with strong AO fluorescence enhancement (Turn-ON). The subsequent addition of a biothiol, like cysteine (Cys), to the G4 complex decreases the fluorescence, as the Pb^2+^ ions released from the G4 complex have a higher interaction affinity with the sulfur atoms of Cys; this further induces the unwinding of the G4 complex to form LFGr-ssDNA. Finally, Epn was added to this, which intercalates with LFGr-ssDNA to form a G4 complex *via* G–Epn–G, resulting in fluorescence recovery (Turn-ON). Accordingly, the Turn-ON fluorescent probe had subsequent limits of detection of 1.5 and 5.6 nM for Pb^2+^ and Epn, respectively. Hence, the reported NMc-CuTA-based sensing platform has potential applications for the detection of Pb^2+^ and Epn in real samples with good sensitivity and selectivity.

## Abstract 2

In this paper, we propose a fluorescent biosensor for the sequential detection of Pb^2+^ ions and the cancer drug epirubicin (Epn) using the interactions between label-free guanine-rich ssDNA (LFGr-ssDNA), acridine orange (AO), and a metal–phenolic nanomaterial.

## Graphical abstract

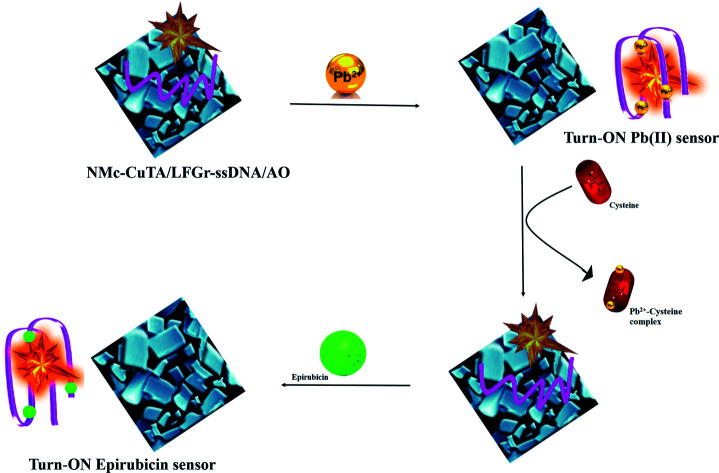

## 1. Introduction

The detection of toxic heavy metal ions based on sensitivity and selectivity criteria has been recognized as an issue for decades worldwide. Pb^2+^ ions are specifically the most toxic heavy metal, causing dangerous health disorders in adults and infants. Even low concentrations of Pb^2+^ consumption have adverse effects, such as hypertension and cardiovascular, reproductive, nervous system, and developmental disorders. The US Environment Protection Agency (EPA) has set the safe limit of Pb^2+^ in drinking water as 72 nM.^1–3^ Epirubicin (Epn) is an anticancer chemotherapeutic drug that exerts cytotoxic effects *via* inhibiting DNA synthesis and DNA replication. Thus, it finds applications in treating various cancer disorders, primarily breast, ovarian, gastric, and lung cancer, but its application is limited as it has serious side effects, like cardiotoxicity, bone marrow suppression, hair loss, low blood counts, and vomiting, when its dosage is high^4,5^. Usually, Pb^2+^ and Epn cannot be identified based on color, taste, or odour. Therefore, it is very essential for researchers to develop sensitive and rapid detection techniques to measure even trace amounts of Pb^2+^ and Epn in biological samples.

Several analytical methods, including atomic absorption spectroscopy, high-performance liquid chromatography (HPLC), electrochemical sensors, cold vapor atomic absorption spectroscopy, and inductively coupled plasma mass spectrometry, have been used for Pb^2+^ and Epn drug analysis in the laboratory.^6–11^ These described techniques often rely on costly instruments, are time-consuming and operationally tedious, and involve sample pretreatment processes; therefore, it is essential to establish appropriate methodology to overcome these disadvantages. The use of fluorescent biosensors is one of the best analytical tools compared with other techniques, as it involves very high intrinsic sensitivity, rapid analysis times, and simplicity. Currently, many researchers are focusing on the design of fluorescence-based biosensors using label- and label-free DNA, which binds to specific analytes with unique recognition.^12–14^ Based on this, large numbers of nanomaterials have been synthesized for the detection of toxic metal ions and biomolecules.

Upconverting nanoparticles (UCNPs), silicon nanocomposites (SiNCs), graphene-like 2D materials, graphene oxide (GO), transition metal dichalcogenides (TMD), graphitic carbon nitride, and transition metal oxides (TMOs)^15–21^ have attracted great attention as transduction elements and supporting substrates in a wide variety of biosensing technologies. 2D materials can provide an extremely high density of active surface sites over a large surface area, making them ideal for biochemical sensing. Although these nanomaterials have been successfully used for the sensitive and specific fluorescence detection of targets, there are some disadvantages, including the non-facile preparation, difficulties in uniform dispersion, and toxicity of some of these nanomaterials. Therefore, it can be anticipated that the search for new nanomaterials is aimed towards replacements.

Metal–organic frameworks (MOFs)^3^ are a novel class of crystalline material. They can show remarkable properties, such as large surface areas, structure tunability, and good aqueous dispersibility. Nevertheless, it cannot be ignored that MOFs still have some defects, restricting their application to a great extent. For instance, MOFs exhibit inferior performance in terms of electrical conduction and have poor stability. Encouragingly, there has been the idea of assembling MOFs with polymer materials to generate highly stable MOF-based bio-polymerized materials, called metal–phenolic nanomaterials (MPNs), which can combine the advantages of both parent materials. Since MPNs are superior to MOFs synthesized *via* traditional methods, they can be excellent alternatives to single MOFs to overcome the above-mentioned difficulties. MPNs have unique surface properties, including the ability to tune the particle size, surface groups, charges, structure, and central metal species. Moreover, MPNs exhibit strong π–π stacking interactions (between exposed nitrogenous nucleobases and the phenolic rings of MPNs)^22^ and coordination interactions (between the DNA phosphate backbone and unsaturated metal sites of MPNs),^23,24^ highly enhancing the sensing performance.

Thus, we focused on the synthesis of a novel monoclinic-like metal–phenolic nanomaterial (MPN), which is a polymeric material involving tannic acid (TA). It has received increasing attention due to its high fluorescence quenching abilities, high surface area, tailorable composition, and diverse advantages, which include biological compatibility, sensing abilities, and environmental applications. Polyphenol is naturally present in vegetables, fruits, *etc.* TA is composed of a central glucose core surrounded by covalently linked digalloyl ester groups with a huge amount of phenolic hydroxyl groups.^25,26^ The preparation of NMc-CuTA involves the use of a plant-based polyphenol (TA) as organic ligands and a metal species (Cu^2+^). The polydentate ligand TA could be easily coordinated with metal ions (Cu^2+^) to form a metal–TA (Cu^2+^–TA) complex. The surface of NMc-CuTA is rich in sp^2^-hybridized carbon atoms and Cu^2+^ ions with a paramagnetic quenching nature. This aspect is beneficial for increasing the π–π and metal–ligand interactions between LFGr-ssDNA, AO, and NMc-CuTA. Therefore, LFGr-ssDNA and AO can be adsorbed on the NMc-CuTA surface *via* π–π stacking and electrostatic interactions but, fortunately, double-stranded DNA (ds-DNA) and G-quadruplexes (G4 complexes) cannot be adsorbed onto NMc-CuTA due to the high shielding of nucleobases within the negatively charged phosphate backbone of dsDNA and G4 complexes. Based on these properties, NMc-CuTA has been utilized to develop a new sensing system for the detection of heavy metal ions and biomolecules.

In this work, we report a novel NMc-CuTA- and LFGr-ssDNA-based fluorescent biosensor with superior sensitivity using the formation of NMc-CuTA/LFGr-ssDNA/AO for sensing Pb^2+^ and the drug Epn. AO is chosen as an excellent fluorescent indicator because this dye emits strong fluorescence in solution. LFGr-ssDNA and AO bind to the surface of NMc-CuTA *via* stacking interactions. At the same time, when Pb^2+^ ions are introduced to the NMc-CuTA/LFGr-ssDNA/AO complex, then LFGr-ssDNA is involved in forming G4 complexes and evacuates from the surface of NMc-CuTA, leading to enhanced AO fluorescence intensity. However, the further addition of Cys, which is a highly effective reactant towards Pb^2+^, results in the formation of a Pb^2+^–Cys complex, which leads to free LFGr-ssDNA and AO. Now, this free LFGr-ssDNA and AO can again be adsorbed onto the NMc-CuTA surface, causing the fluorescence signal to decrease. When Epn is introduced into the quenched solution, it strongly intercalates with LFGr-ssDNA to form a G4 complex once again, which leads to the enhancement of the fluorescence intensity of AO. Thus, a novel Turn-ON fluorescence-signal-based label-free biosensor for the sequential detection of Pb^2+^ and Epn was developed, and this has not been reported by any other researchers to date.

## 2. Experimental section

### 2.1. Materials and methods

Tannic acid (TA) and copper nitrate trihydrate were purchased from Marcklin Biochemical Co., Ltd. Pluronic F127 was purchased from Sigma-Aldrich. The Pb^2+^ and Epn molecule binding DNA probe was synthesized using integrated DNA technology. The following DNA sequence was used: 5′-CCTGGGCGGGTAGGGCGGGATCGGGTCCAGGT. A stock solution of DNA was prepared *via* dissolving 50 mM DNA in Tris hydrochloride (Tris–HCl) buffer solution (pH = 7.5) and it was stored at 4 °C. Epirubicin, lead nitrate, mercury nitrate, calcium chloride, nickel chloride, cobalt chloride, copper chloride, barium chloride, iron chloride, magnesium chloride, manganese chloride, imatinib (IMT), ampicillin (AMP), streptomycin (STR), tamoxifen (TMF), and other standard chemicals were purchased from Sigma-Aldrich (India) and SRL Pvt Ltd (India). The stock buffer and metal salt solutions used in the experiments were prepared using double-distilled water.

### 2.2. Structural characterization

The fluorescence emission spectra were measured using a HORIBA JOBIN YVON Fluoromax-4 spectrofluorometer with a xenon lamp excitation source at excitation and emission wavelengths of 490 and 530 nm, respectively, and a slit width of 5 nm. The morphological appearance of NMc-CuTA was studied based on surface images obtained using a high-resolution scanning electron microscope (HRSEM, Nanosem 430) and high-resolution transmission electron microscope (HRTEM) at a voltage of 200 kV with JEOL/JEM-2100 apparatus. Element mapping was also conducted with a Nanosem 430 HRSEM equipped with an energy dispersive X-ray spectrometer (EDX) (ISIS300; Oxford) to identify the distributions of Cu^2+^ ions, carbon, and oxygen on the surface of NMc-CuTA. An Agilent Technologies FT-IR spectrometer (USA) was used to record the Fourier-transform infrared (FT-IR) spectra *via* the KBr pellet technique. FT-IR spectroscopy was used to investigate the functional groups on the surface of NMc-CuTA in the range of 400–1400 cm^−1^. X-ray diffraction (XRD) spectra were obtained using a PAN analytical X'pert pro X-ray diffractometer with Cu Kα radiation. XRD patterns were used to find out the crystalline nature of the synthesised NMc-CuTA, and they were recorded in the 2*θ* range of 10–80°. X-ray photoelectron spectroscopy (XPS, PerkinElmer Phi 1600 ESCA system), using Mg (1486.6 eV) as the radiation source, was used to estimate the surface element composition, element binding configuration, and charge compensation of NMc-CuTA.

### 2.3. Analysis of real clinical samples (RCS)

For the quantitative analysis of Pb^2+^ and Epn in RCS, urine samples were filtered through a membrane to remove all insoluble impurities. Aliquots of the urine samples were spiked with a stock solution of Pb^2+^ and Epn diluted with Tris–HCl buffer. Then, fluorescence measurements were performed.

### 2.4. Procedure for Pb^2+^ and Epn detection

The detailed procedure for the detection of Pb^2+^ and Epn is as follows. Initially, the incorporated reaction was performed by mixing 500 μL of 300 nM LFGr-ssDNA and AO, each made with Tris buffer (pH 7.5), followed by the addition of 30 μg mL^−1^ NMc-CuTA into a micro-centrifuge tube. After 20 min of reaction, the solution was transferred into a cuvette and fluorescence spectra were measured at excitation and emission wavelengths of 490 and 530 nm, respectively, showing a fall in AO fluorescence due to the quenching behavior of NMc-CuTA. Later, freshly prepared Pb^2+^ solution at the desired concentration was added and incubated for 30 min at 35 °C, leading to the formation of a G4 complex, which results in the enhancement of the fluorescence intensity of AO. Furthermore, this sensing strategy was prolonged for the subsequent detection of Epn *via* the elimination of Pb^2+^ using Cys, leading to the turn-off of the fluorescence signal. Finally, the desired concentration of Epn was added into the above solution, which was incubated for 30 min at 35 °C, again leading to the formation of the G4 complex and causing an increase in AO fluorescence intensity.

### 2.5. Synthesis of NMc-CuTA

**Scheme 1:**
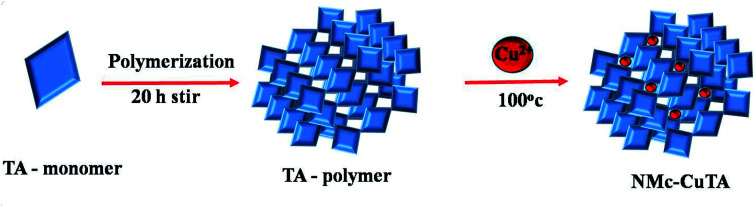
A schematic representation of the synthesis of NMc-CuTA.

The novel monoclinic nanomaterial was synthesized according to the literature with some modifications. NMc-CuTA was prepared *via* a metal–ligand-coordination-driven self-assembly process.^27^ Briefly, 500 mg of F127 was dissolved in a mixture of water (5 mL) and ethanol (10 mL). Then, 900 μL of ammonia was slowly added followed by the addition of tannic acid (500 mg), and the above solution was magnetically stirred for 20 h. To this, 250 mg of Cu(NO_3_)_2_·3H_2_O dissolved in a minimum volume of water was added, followed by stirring for 24 h. The formed product was transferred into a pressure tube and placed in an oil bath for 15 h at 100 °C. Further, the solution was centrifuged and washed several times with water and ethanol to remove unreacted materials. Finally, the obtained NMc-CuTA material was dried at 70 °C for 7 h, with the synthesis process shown in Scheme 1.

## 3. Results and discussion

### 3.1. Characterization

**Fig. 1:**
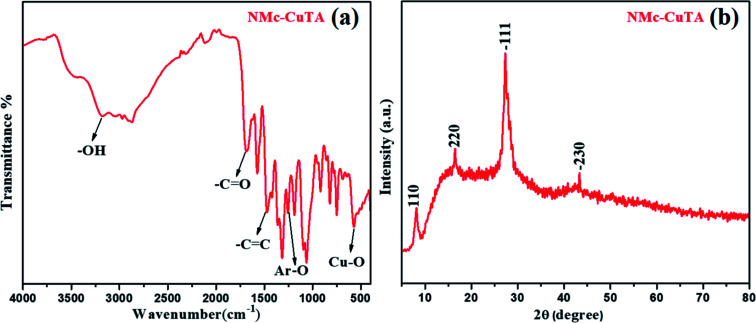
(a) FT-IR and (b) XRD spectra of NMc-CuTA.

In order to confirm the structural transformation of the prepared NMc-CuTA, a few studies were carried out. Initially, FT-IR spectral studies were done, and the results are shown in Fig. 1a. It is shown that the high intensity peak at 3172 cm^−1^ corresponds to the typical –OH stretching frequency, the peak at 3080 cm^−1^ can be assigned to –C–H aromatic ring stretching, and the peak at about 1693 cm^−1^ is due to the C=O stretching of the carbonyl group. Further, the specific absorption peak located at 1470 cm^−1^ was attributed to C=C aromatic ring stretching, and the peak at 1270 cm^−1^ corresponds to Ar–O (phenolic) groups. Furthermore, the peak appeared below 600 cm^−1^ is attributed to Cu–O vibrations; this clearly indicates successful bonding with copper atoms on the surface of tannic acid.^28,29^ The –OH frequency reduction from 3500 cm^−1^ to 3172 cm^−1^ gives clear evidence that tannic acid will form a complex with copper metal ions. The above results indicate the presence of numerous –C=C and phenolic groups on the surface of NMc-CuTA along with covalently bonded copper metal ions. The above fact suggests that the prepared NMc-CuTA complex can show greatly enhanced hydrophilicity and stability in aqueous solution. As shown in Fig. 1b, the XRD diffraction peaks located at 2*θ* values of about 8.2, 16.4, 27.1, and 43.0° could be ascribed to the (110), (220), (−111), and (−230) crystal planes, respectively, which might suggest a monoclinic copper–tannic acid lattice. In particular, the peak at a 2*θ* value of 43.0° was assigned to the copper (−230) plane (PDF no. 00-032-0331).^30^

**Fig. 2:**
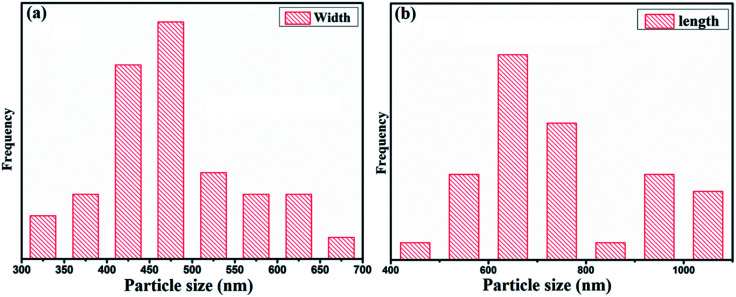
Particle sizes of NMc-CuTA: (a) width and (b) length.

**Fig. 3:**
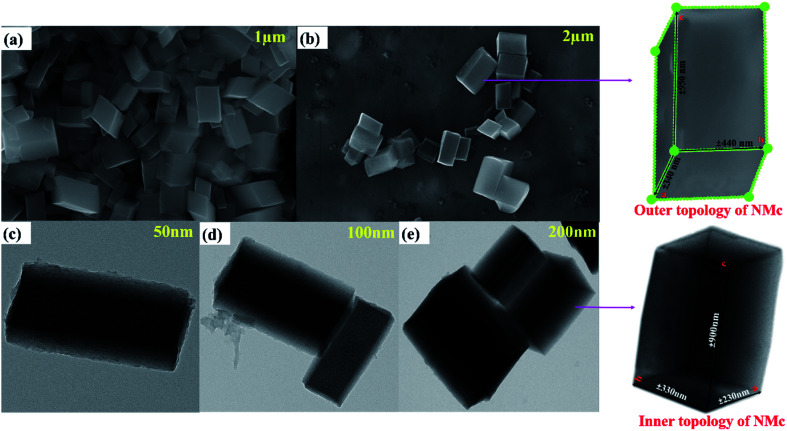
(a and b) HRSEM and (c–e) HRTEM images of NMc-CuTA.

The morphology and elemental analysis of the synthesized metal–phenolic (copper–tannic acid) nanomaterial were studied *via* SU-70 high-resolution scanning electron microscopy along with X-ray energy dispersive spectrometry (EDS). The HRSEM image confirms that the synthesized copper–tannic acid particles possess a monoclinic shape (Fig. 3a and b). Thus, the material was named “nano-monoclinic copper tannic acid” (NMc-CuTA). As NMc-CuTA has a large surface area, it can adsorb LFGr-ssDNA and AO efficiently due to π–π stacking, which in turn quenches the fluorescence of AO due to the paramagnetic quenching abilities of the Cu^2+^ ions. HRSEM images show that typical NMc-CuTA particles have a width of ∼450 nm and a length of ∼650 nm, as shown in Fig. 2a and b.

**Fig. 4:**
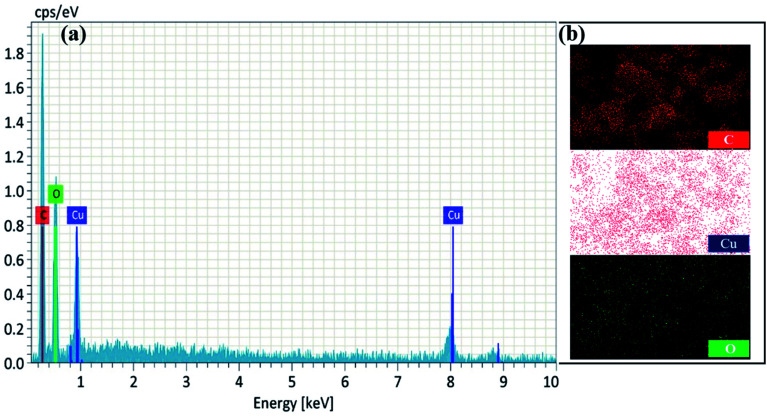
EDX spectrum (a) and elemental mapping (b) of NMc-CuTA.

Furthermore, a clear inner surface investigation of the as-prepared nanomaterial was carried out *via* HRTEM, as shown in Fig. 3c–e. HRTEM images show that the lengths of the unit cell parameters obtained were in the order of *a* ≠ *b* ≠ *c* (*a* ± 230 nm; *b* ± 330 nm and *c* ± 900 nm), which clearly expresses that the Cu^2+^ ions are coordinated to the surface of tannic acid with monoclinic geometry. The EDX spectrum and elemental mapping of NMc-CuTA are shown in Fig. 4a and b. The EDX spectrum confirms the presence of the elements carbon (C), oxygen (O), and copper (Cu) with atomic percentages of 45, 50, and 5, respectively. This result is consistent with the XPS data. The elemental mapping of this nanomaterial also reveals the presence of the elements C, O, and Cu, marked in different colours. The elemental mapping and EDX results conclude that the synthesized nanomaterial was found to be free from any other elements or impurities.

**Fig. 5:**
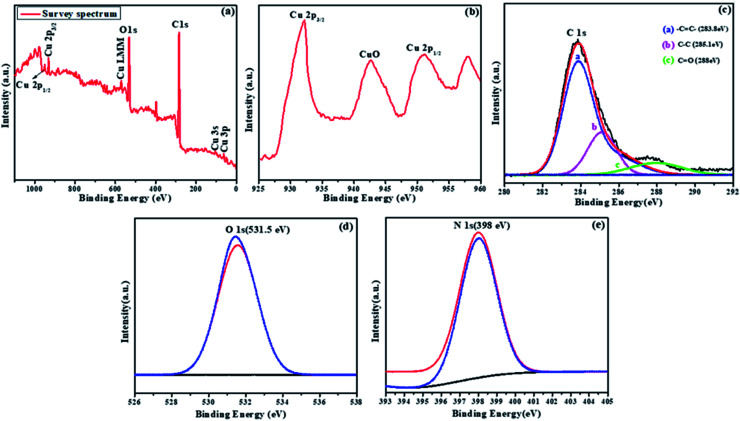
XPS spectra of NMc-CuTA: (a) survey, (b) Cu 2p, (c) C 1s, (d) O 1s, and (e) N 1s spectra.

To confirm the purity and elemental composition of the prepared NMc-CuTA material, X-ray photoelectron spectroscopy (XPS) measurements were performed, as seen in Fig. 5a–e. As shown in Fig. 5a, the spectrum of NMc-CuTA confirmed the presence of C 1s, O 1s, N 1s, and Cu 2p peaks, without any impurities. The peaks found at 950.1 and 932.1 eV in the Cu 2p XPS spectrum are attributed to Cu 2p_1/2_ and Cu 2p_3/2_, respectively (Fig. 5b). The high-resolution C 1s spectrum was fitted with three peaks at about 283.8, 285.1, and 288 eV, which could be assigned to C=C, C–C, and C=O or C–O, respectively, with relative amounts of 48%, 30%, and 12%, respectively^31^ (Fig. 5c). The O 1s XPS spectrum (Fig. 5d) exhibits a peak at 531.5 eV, which was assigned to the lattice oxide oxygen of the metal oxide, indicating the formation of metal–oxygen (Cu–O) bonds between copper and tannic acid. The N 1s XPS spectrum has a single intense peak at 398 eV, which corresponds to the N atoms of ammonia (Fig. 5e). Therefore, with the help of XPS data, we conclude that NMc-CuTA has ionic copper and a large amount of sp^2^-hybridized carbon; it can effectively diminish the fluorescence properties of AO and facilitate the adsorption of LFGr-ssDNA on its surface *via* π–π stacking interactions.

N_2_ adsorption–desorption studies are used to find the surface area of NMc-CuTA, and results are shown in Fig. S1.† The BET surface area and pore volume of NMc-CuTA are found to be 178.692 m^2^ g^−1^ and 0.299 cm^3^ g^−1^, respectively. From these observations, polyphenol-derived NMC-CuTA is found to have a high surface area and pore volume. Therefore, NMC-CuTA had high adsorption abilities toward ssDNA and AO due to its high surface area.

### 3.2. The quenching behavior of NMc-CuTA and confirmation studies

**Fig. 6:**
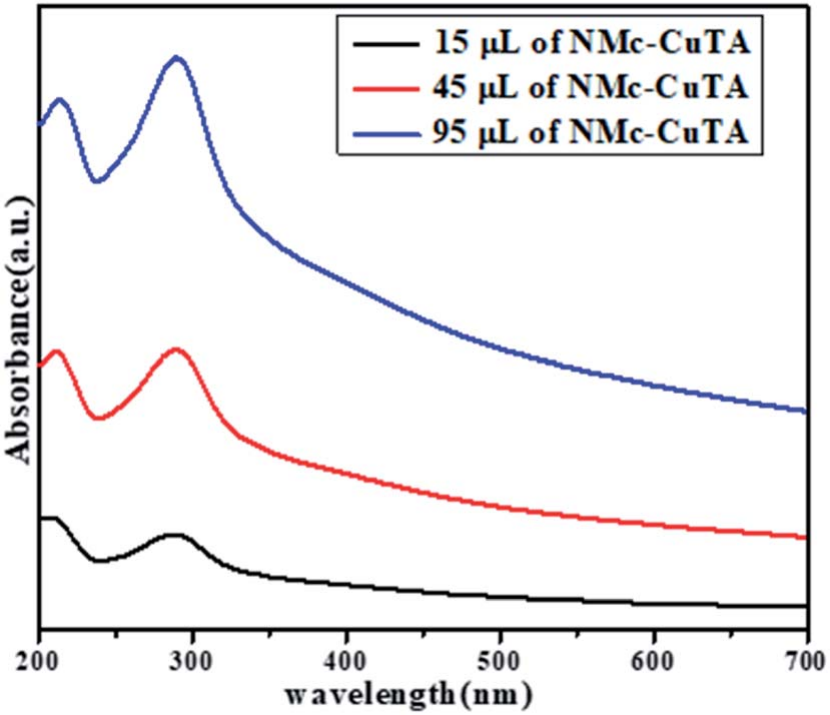
UV-spectra of different amounts of NMc-CuTA.

The optical properties of NMc-CuTA were investigated *via* UV-vis spectroscopy in the range of 200–800 nm. In general, the UV spectrum of tannic acid shows two absorption peaks at 210 and 280 nm, which are attributed to π–π* and n–π* transitions, respectively,^32^ and the Cu^2+^ ion absorption peak is found around 275 nm. Due to bond formation between copper and tannic acid, the absorption peaks at 280 and 275 nm merge to give a new absorption peak at 290 nm, as shown in Fig. 6.

**Fig. 7:**
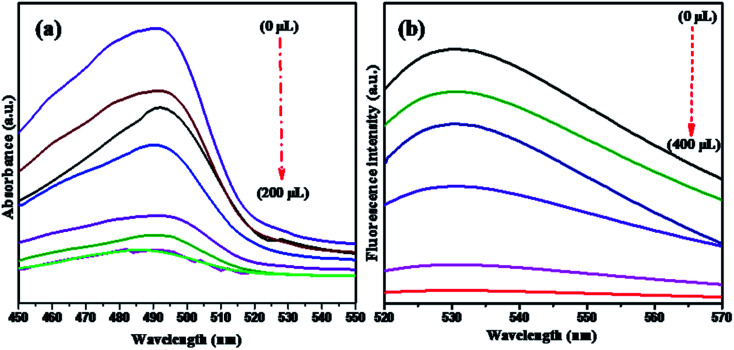
(a) UV-visible spectra and (b) fluorescence spectra showing the quenching of AO in the presence of various amounts of NMc-CuTA. (a) AO treated with 0, 50, 75, 100, 150, 175, 200, and 225 μL of NMc-CuTA; and (b) AO treated with 0, 50, 100, 200, 300, and 400 μL of NMc-CuTA.

NMc-CuTA has good fluorescence quenching abilities, as shown in Fig. 7. The UV absorption spectrum of AO shows a strong absorption band at 490 nm. The absorption band intensity decreases with respect to the amount of NMc-CuTA (Fig. 7a). As the absorption band has high hypochromicity, we conclude that AO and NMc-CuTA undergo strong static interactions due to the large amount of sp^2^-hybridized carbon atoms. This may also be due to the paramagnetic quenching characteristics of copper(ii) ions present on the surface of NMc-CuTA.

The fluorescence emission spectra of AO in the presence and absence of NMc-CuTA were studied. As the amount of NMc-CuTA is increased, the fluorescence emission intensity of AO gradually decreases due to intermolecular energy transfer between AO and NMc-CuTA. This action requires large overlap between the emission spectrum of the energy donor AO and the absorption spectrum of the energy acceptor NMc-CuTA, as this is a basic necessity for the FRET process. Thus, the mechanism behind the strong fluorescence quenching effect of NMc-CuTA toward AO could be concluded, as shown in Fig. 7b.

### 3.3. The sensing mechanism toward Pb^2+^ ions and Epn

**Scheme 2:**
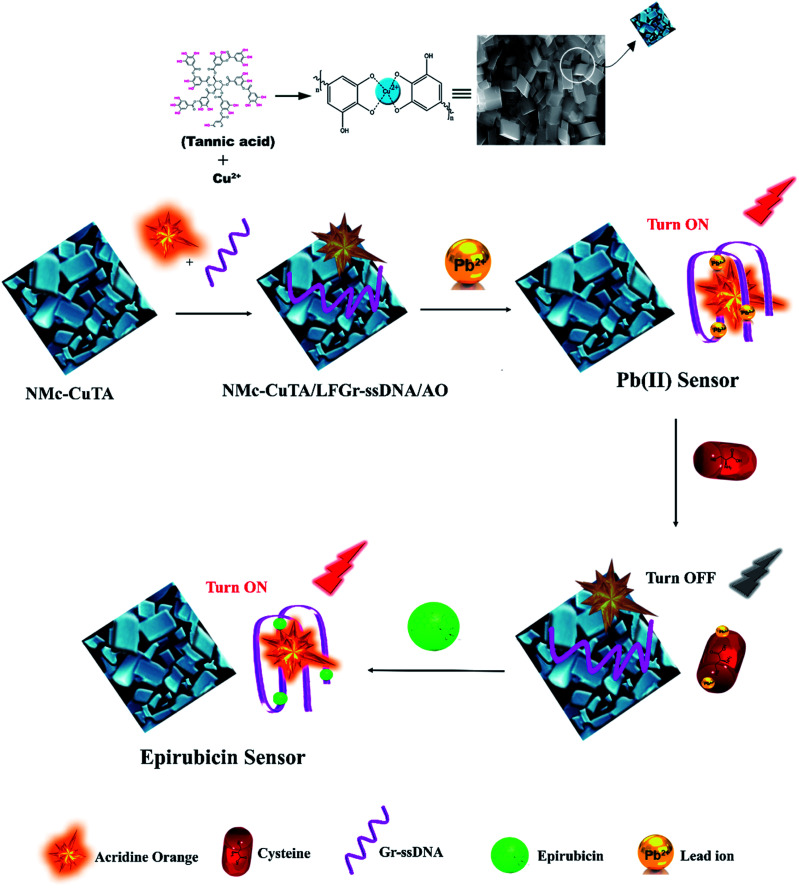
A schematic illustration of the fluorescence detection of Pb^2+^ ions and the anti-cancer drug Epn using the NMc-CuTA/LFGr-ssDNA/AO sensing platform.

**Fig. 8:**
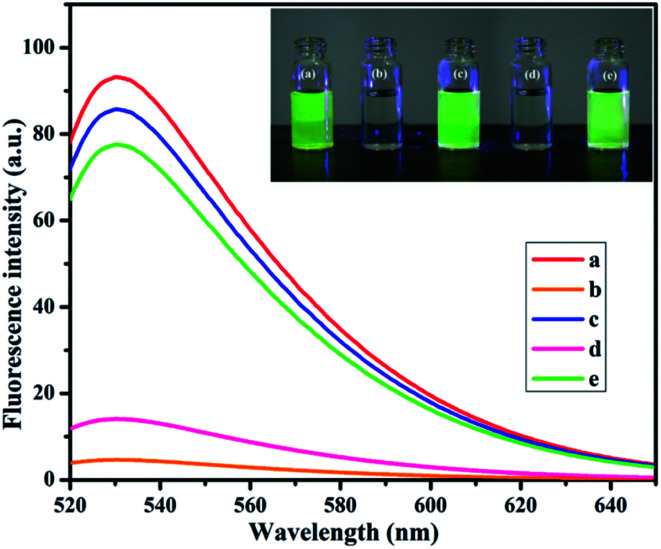
The fluorescence emission spectra and UV properties (inset) of AO alone (a), the NMc-CuTA/LFGr-ssDNA/AO complex (b), the NMc-CuTA/Pb^2+^–AO–G4 complex (c), the NMc-CuTA/AO/LFGr-ssDNA/Pb^2+^–Cys complex (d), and the NMc-CuTA/Epn–AO–G4 complex/Pb^2+^–Cys complex (e).

In the designed strategy, the mechanism proposed allowed a Turn-ON fluorometric biosensor for Pb^2+^ and Epn with superior sensitivity *via* the quenching of the AO fluorophore by the NMc-CuTA/LFGr-ssDNA complex, as shown in Scheme 2. The single-stranded DNA was designed with a large extended G-rich sequence, which is utilized as the specific target analyte. LFGr-ssDNA binds to the surface of NMc-CuTA through π–π stacking and electrostatic interactions. AO is an excellent fluorescent indicator because this dye emits strong fluorescence in solution (Fig. 8a). The fluorescence of AO could be quenched with NMc-CuTA in end-stacking mode to form the NMc-CuTA/LFGr-ssDNA/AO complex, as shown in Fig. 8b, resulting in a significant decrease in the AO fluorescence signal due to the paramagnetic quenching nature of Cu^2+^*via* fluorescence resonance energy transfer (FRET) from the fluorophore singlet excited state (S_n_) to the paramagnetic Cu^2+^ center. At the same time, Pb^2+^ ions introduced into the NMc-CuTA/LFGr-ssDNA/AO complex form a Pb^2+^–G4 complex, leading to enhanced fluorescence intensity (Fig. 8c) because the G4 complex strongly captures AO from the surface of NMc-CuTA. The further addition of cysteine, as a highly effective reactant towards Pb^2+^, results in the formation of Pb^2+^–Cys complexes, leading to the generation of free LFGr-ssDNA and AO, which again are adsorbed onto the NMc-CuTA surface with a reduced fluorescence signal in turn. When Cys is added to the AO/Pb^2+^–G4 complex, it efficiently attracts Pb^2+^ ions from the G4 complex to form more stable Pb^2+^–Cys complexes, as biothiol-like Cys contains active functional groups such as –SH, –NH_2_, and –COOH. These groups strongly react with Pb^2+^ (based on HSAB theory), resulting in the precipitation of PbS or complexation *via* covalent forces, electron sharing, or exchange between the active functional group and Pb^2+^ ions. In the absence of Pb^2+^ ions, the G4 complex is unstable, so it aligns itself with unstructured DNA. Due to the absence of the G4 complex in solution, AO itself gets adsorbed on the NMc-CuTA surface, which significantly decreases the fluorescence intensity of AO, as shown in Fig. 8d. When epirubicin (Epn), an anti-cancer drug, is added to the above solution, it strongly intercalates with the G-bases of unstructured DNA. This intercalation leads to the formation of hydrogen and covalent bonds between Epn and DNA guanine base pairs. These interactions can be stabilized by a covalent bond mediator, say cellular formaldehyde, resulting in the formation of Epn–G4 complexes, which can also capture AO from the surface of NMc-CuTA, leading to fluorescence signal recovery (Fig. 8e).

### 3.4. Analysis of CD spectra

**Fig. 9:**
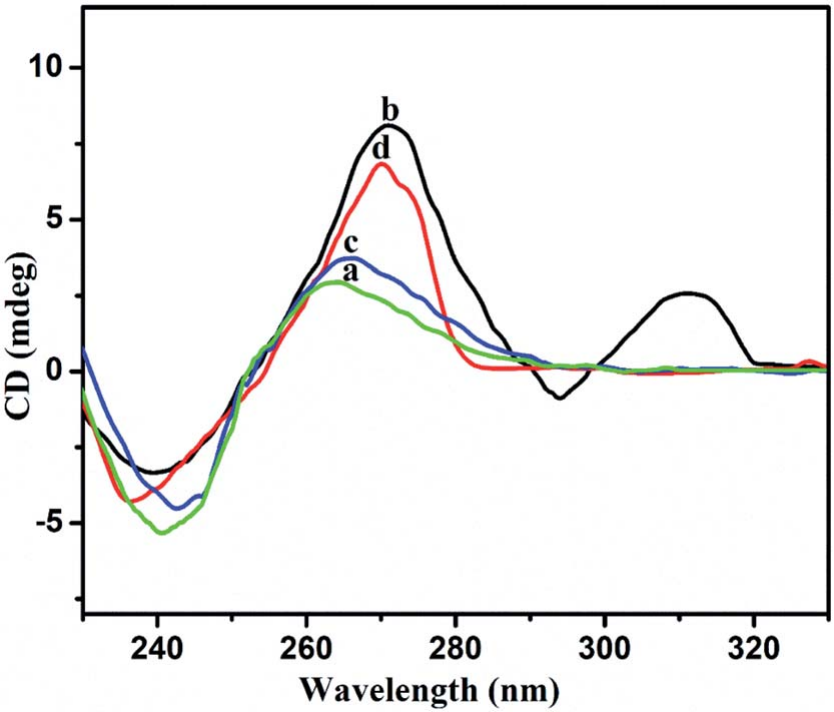
CD spectra for Gr-ssDNA (3 μM) (a); Gr-ssDNA (3 μM) with Pb^2+^ (5 μM) (b); Gr-ssDNA (3 μM) with Pb^2+^ (5 μM) and Cys (5 μM) (c); and Gr-ssDNA (3 μM) with Pb^2+^ (5 μM), Cys (4 μM), and Epn (5 μM) (d).

The structural changes were further analyzed *via* circular dichroism (CD) spectroscopy. CD is a method that is quite sensitive to the confirmations of chiral structures, nucleic acids, and G-quadruplex structures. CD spectral analysis for 3 μM Gr-ssDNA in the absence and presence of Pb^2+^ and Epn is shown in Fig. 9. In the absence of Pb^2+^ and Epn, the CD spectrum has a low amplitude, as ssDNA has a barely chiral structure (Fig. 9, curves a and c). When incubated in 5 μM Pb^2+^, the spectrum possess a positive CD peak near 311 nm coupled with a negative peak at 294 nm, which are characteristic of an antiparallel G4 structure orientation (Fig. 9, curve b).^33^ On the other hand, a confirmational change of ssDNA occurs upon the addition of 5 μM Epn, and the CD spectrum shows a positive peak at 270 nm and a negative peak at 236 nm (Fig. 9, curve d). These two peaks correspond to the parallel orientation of the G4 structure. Based on these results, we can conclude that Epn strongly intercalates with ssDNA,^20^ which leads to the formation of Epn-stabilized parallel G4 structures.

### 3.5. Optimization of the detection conditions

**Fig. 10:**
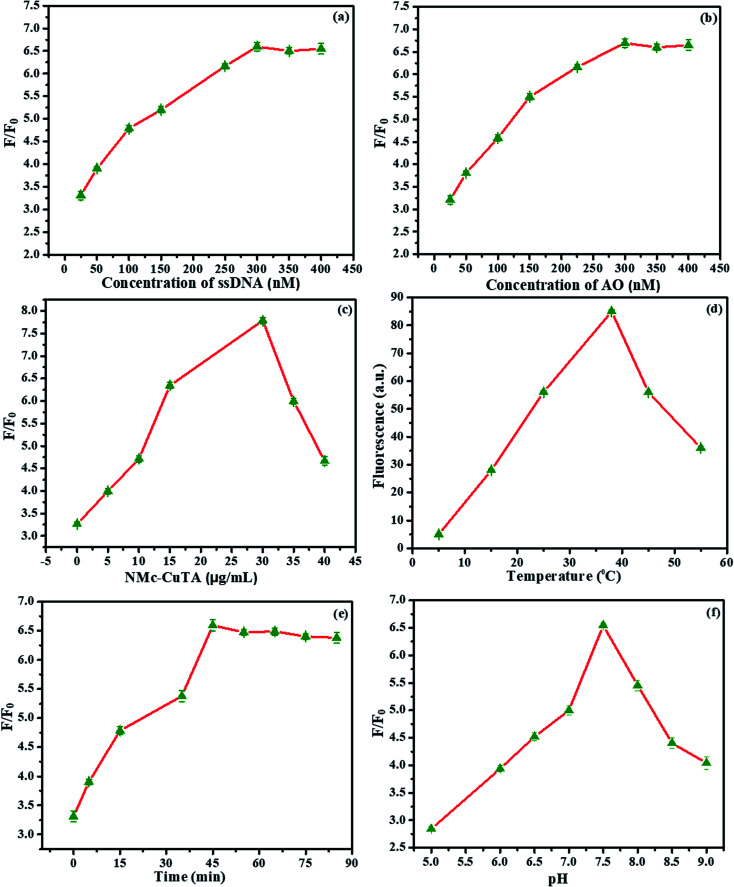
Optimization of the detection conditions. (a) The effects of DNA concentration on fluorescence (different concentrations of LFGr-ssDNA were mixed with 30 μg mL^−1^ NMc-CuTA, 300 nM AO, and Pb^2+^ (1 μM) or Epn (1 μM) in 20 nM Tris buffer). (b) The effects of AO on fluorescence (solutions of 20 mM Tris buffer containing various concentrations of AO with LFGr-ssDNA (300 nM), 30 μg mL^−1^ NMc-CuTA, and Pb^2+^ (1 μM) or Epn (1 μM) were used). (c) The fluorescence recovery abilities of NMc-CuTA (various amounts of NMc-CuTA, 300 nM AO, LFGr-ssDNA (300 nM), and Pb^2+^ (1 μM) or Epn (1 μM) were mixed in 20 mM Tris HCl buffer (pH = 7.5), and then the AO fluorescence emission intensity was recorded). The effects of (d) temperature, (e) time, and (f) pH on the fluorescence recovery (20 mM Tris–HCl buffer solutions containing AO (300 nM), LFGr-ssDNA (300 nM), NMc-CuTA (30 μg mL^−1^), and Pb^2+^ (1 μM) or Epn (1 μM) were used, with fluorescence spectra measured at different temperatures, times, and pH levels).

The optimization of the proposed sensor was evaluated based on various influencing factors, which include the concentrations of LFGr-ssDNA and AO, the amount of NMc-CuTA, and the effects of the reaction time, pH, and temperature on the signal-to-background ratio (S/B ratio), as shown in Fig. 10. Initially, the AO fluorescence intensity of the sensing system was studied *via* varying the concentration of LFGr-ssDNA, because the amount of G4 complex formation depends on the concentration of LFGr-ssDNA. The AO emission intensity was enhanced to a maximum level due to an increasing amount of G4 complex formation with respective analytes (Pb^2+^ and Epn); therefore, the AO fluorescence emission indirectly depends on the concentration of LFGr-ssDNA. Accordingly, the concentration of LFGr-ssDNA was optimized between 25 and 400 nM, (Fig. 10a); the experimental results clearly show that fluorescence emission reached the maximum level when the concentration of LFGr-ssDNA was 300 nM. Therefore, the optimal concentration of LFGr-ssDNA (300 nM) was chosen as the fixed concentration for further studies. Similarly, the fluorophore concentration was optimised between 25 and 400 nM (Fig. 10b). The experimental results show that the SBR of the AO signal reached a maximum with 300 nM AO. Therefore, 300 nM AO was chosen as the optimized concentration for this sensing system. Further, the amount of NMc-CuTA needs to be optimised, as the nanomaterial has the ability to quench the fluorescence intensity of AO. Similarly, the amount of NMc-CuTA was investigated in the presence of 300 nM LFGr-ssDNA and 300 nM AO. The experimental results show that the SBR of the AO signal decreases with an increasing amount of NMc-CuTA, and the AO signal intensity reached a minimum at an optimal concentration of 30 μg mL^−1^; this also proves to be the optimal concentration for fluorescence recovery, as shown in Fig. 10c. Therefore, 30 μg mL^−1^ was chosen as the optimised concentration for further studies.

The influence of the reaction temperature and incubation time on the performance of the sensing system was also optimized (Fig. 10d and e). The SBR of AO fluorescence increased to a maximum at 35 °C. The incubation time was monitored from 0 to 90 minutes. At an incubation time of 45 min, the fluorescence intensity reached a maximum value, so we chose 45 min as the optimal fluorescence recovery time. Therefore, further experiments were all conducted for 45 min at 35 °C for the detection of Pb^2+^ and Epn. Moreover, the influence of pH on the SBR of the AO signal was also optimized (Fig. 10f). The optimal pH was studied by using buffer solutions with different pH values; based on the results, a pH value of 7.5 was suitable for this sensing system.

### 3.6. Sensitivity toward Pb^2+^ and Epn

**Fig. 11:**
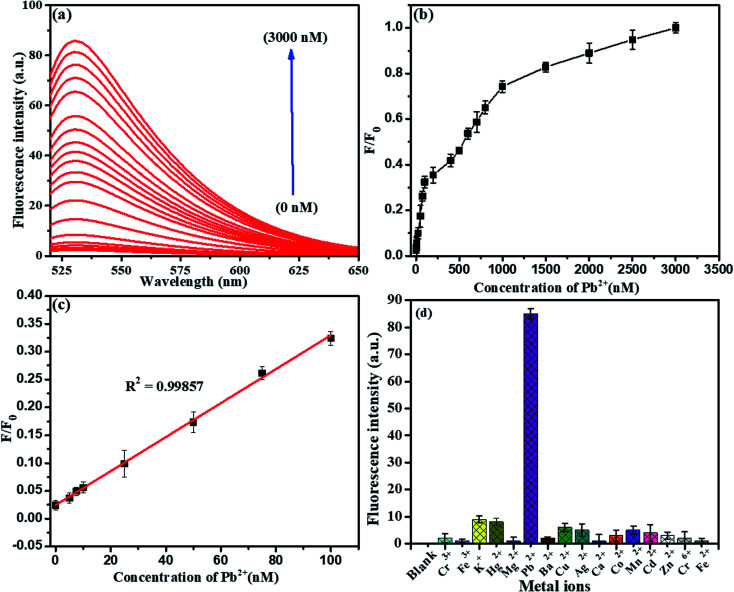
(a) Fluorescence emission spectra of the optimized sensor in response to different concentrations of Pb^2+^: 0, 5, 7.5, 10, 25, 50, 75, 100, 200, 400, 500, 600, 700, 800, 1000, 1500, 2000, 2500, and 3000 nM. (b) Integrated fluorescence response *versus* the Pb^2+^ concentration. (c) The linear relationship between the fluorescence emission intensity and Pb^2+^ concentration. (d) The selectivity of the optimized fluorescent sensor for Pb^2+^ (500 nM) in comparison to other metal ions (1000 nM). The error bars display the standard deviations from three individual tests.

To evaluate the presence of Pb^2+^ ions, a fluorescence spectra study was chosen. It was found that the fluorescence intensity was directly proportional to the concentration of detected Pb^2+^ ions using the above standard optimized conditions, as shown in Fig. 11. Herein, we explored the LFGr-ssDNA-based fluorescent Pb^2+^ sensor with improved sensitivity using AO dye adsorbed on the surface of NMc-CuTA with the LFGr-ssDNA complex. AO dye was chosen as the fluorescent indicator because this dye emits strong fluorescence in solution. In the absence of Pb^2+^ ions, there is no fluorescence signal because the fluorescence intensity was completely quenched by NMc-CuTA. In the presence of Pb^2+^, the fluorescence intensity of the NMc-CuTA/LFGr-ssDNA/AO complex dramatically increased due to the formation of G4 complexes. This remarkable fluorescence intensity increased with an increase in the concentration of Pb^2+^ ions from 0 to 3000 nM, as seen in Fig. 11a and b. The relative fluorescence intensity was plotted against the concentration of Pb^2+^ ions in the range from 0 to 100 nM, and the linear correlation co-efficient (*R*^2^) was 0.9985, as shown in Fig. 11c. The detection limit for Pb^2+^ ions was 1.5 nM, which was calculated based on the 3*σ*/slope rule.

**Fig. 12:**
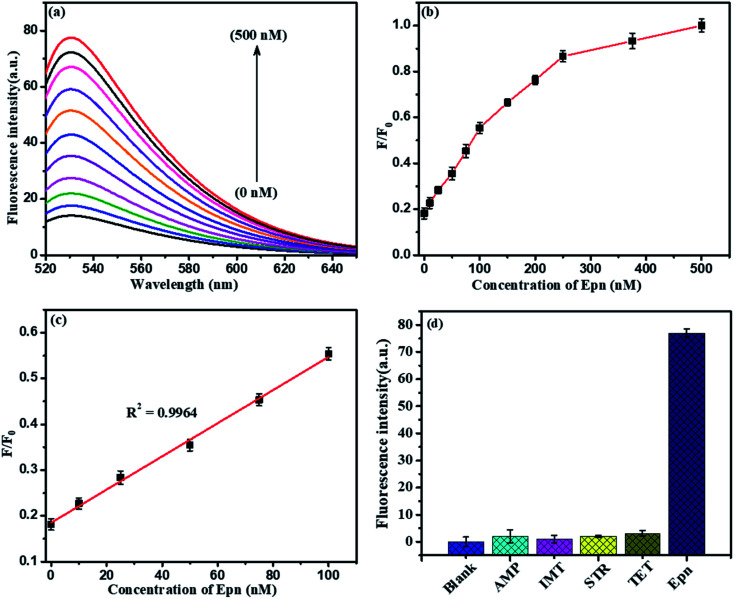
(a) Fluorescence spectra of the optimized sensor in response to different concentrations of Epn: 0, 10, 25, 50, 75, 100, 150, 200, 250, 375, and 500 nM. (b) Integrated fluorescence response *versus* the Epn concentration. (c) The linear relationship between the fluorescence emission intensity and the Epn concentration. (d) The selectivity of the optimized fluorescence sensor for Epn (500 nM) in comparison to other drugs (1000 nM). The error bars display the standard deviations from three individual tests.

Further, the NMc-CuTA/Pb^2+^–G4–AO system acts as another fluorescent assay for Epn detection. At first, 500 nM Cys was added to the above system and it was incubated for 30 min; the Pb^2+^ ions were strongly attracted by Cys to form a Pb^2+^–Cys complex, leading to a significant decrease in the fluorescence signal. Then, the introduced Epn intercalates with the G bases in ssDNA to form G4 complexes, which capture AO; the fluorescence enhancement was monitored in the range of 0 to 500 nM, as shown in Fig. 12a and b. A good linear relationship (*R*^2^ = 0.9964) was observed in the range from 0 to 100 nM and the limit of detection (LOD) was 5.6 nM (Fig. 12c). Furthermore, the proposed Pb^2+^ and Epn sensor showed superior performance compared to other LFGr-ssDNA-based methods. The high sensitivity of the proposed sensor can be attributed to the quenching ability of NMc-CuTA, indicating the formation of NMc-CuTA/LFGr-ssDNA/AO complexes, which efficiently act as sensing probes. The advantages of our proposed sensing probe were compared with earlier reported biosensors, as shown in Tables 1 and 2.

**Table 1:** A comparison of different sensing probes used to determine Pb^2+^ ions^a^

Method	Sensing probe	LOD	Linear range	Real sample	Ref.
FM	T30695/SYBER green	18 nM	0 to 480 nM	Lake water	36
FM	AuNPs on GO	10 nM	50 to 1000 nM	Tap water	37
FM	NMSET/FAM-AuNPs	10 nM	12.5 to 100 nM	Tap water	38
FM	DNA-AgNCs	3 nM	5 to 50 nM	Tap and lake water	39
FM	Aptamer-functionalized UCAuNPs	5.7 nM	25 to 1400 nM	Tea and wastewater	40
FM	NMc-CuTA/LFGr-ssDNA/AO	1.5 nM	0 to 100 nM	Urine	This work

a FM: fluorescence method; LOD: limit of detection; AuNPs: gold nanoparticles; GO: graphene oxide; NMSET: nanomaterial surface energy transfer; AgNCs: silver nanoclusters; UCAuNPs: upconversion gold nanoparticles.

**Table 2:** A comparison of different sensing probes used to determine the drug Epn^a^

Method	Sensing probe	LOD	Linear range	Real sample	Ref.
HPLC/UV	C18 column	8 ng mL^−1^	1.0–100.0 μg mL^−1^	Injection	7
FM	Nd^3+^ (UCNPs)	50 nM	0.09–189.66 μM	Urine	16
ECM	DNA/AuNS/Fe_3_O_4_/DABCO	40 nM	0.07–21 μM	Urine	20
ECM	SWCNT-electrode	20 nM	0.05–10 μM	Injection	41
FM	NMc-CuTA/AO/LFGr-ssDNA	5.6 nM	0 to 100 nM	Urine	This work

a FM: fluorescence method; LOD: limit of detection; ECM: electrochemical method; AuNS: gold nanostructure; DABCO: diazoniabicyclo[2.2.2]octane; SWCNT: single-walled carbon nanotube; UCNPs: upconversion nanoparticles.

### 3.7. Selectivity of Pb^2+^ and Epn

Selectivity is another important issue when assessing the performance of a newly proposed sensor, and the LFGr-ssDNA-based biosensor showed remarkably high selectivity toward Pb^2+^ and Epn (Fig. 11d and 12d). To investigate the selectivity of the NMc-CuTA/LFGr-ssDNA/AO complex, its responses to various metal ions were recorded, as shown in Fig. 11d. From the results, it is clearly shown that 500 nM Pb^2+^ could induce a significant fluorescence enhancement in the sensing system but all other metal ions (Hg^2+^, Cu^2+^, Ag^+^, Ni^2+^, Zn^2+^, Mg^2+^, K^+^, Cr^3+^, Mn^2+^, *etc.*) at concentrations of 1000 nM did not result in an obvious fluorescence enhancement. It should be noted that Pb^2+^ ions form stable G4 complexes, as there are shorter M–O and O–O bonds than those stabilized by K^+^.^34^ The high selectivity of the developed Pb^2+^ sensor may result in different Pb^2+^-stabilized G4 complex structures compared to K^+^-stabilized G4 complex structures. The selectivity of the Epn sensor was evaluated using the NMc-CuTA/LFGr-ssDNA/AO system after Pb^2+^ was successfully eliminated *via* the introduction of Cys. The selectivity of the Epn sensor system was investigated using other common drug biomolecules, such as IMT, AMP, STR, and TMF. The fluorescence intensity was not enhanced upon the addition of other drug biomolecules but it was significantly enhanced during the addition of Epn due to the formation of hydrogen and covalent bonds between Epn and DNA guanine base pairs. These interactions can be stabilized *via* covalent-bond-mediated cellular formaldehyde, resulting in the formation of an Epn–G4 complex.^35^ These results indicate that the system possesses excellent selectivity for epirubicin and no interference from other drug biomolecules was seen (Fig. 12d).

### 3.8. Analysis of Pb^2+^ and Epn in RS

The practical applicability of this (NMc-CuTA/LFGr-ssDNA/AO) sensing strategy was evaluated through the detection of Pb^2+^ and Epn in real samples. Real samples, *i.e.*, urine, were obtained from human subjects, and their consent was recorded through SRM Hospital, Tamil Nadu, India. Before the fluorescence sensing analysis of Pb^2+^ and Epn, all urine samples were pre-treated. 50 mL of sample was filtered through a 0.2 μm Millipore membrane and ultrasonicated for 20 min. Then the filtrate was centrifuged for 20 min at 400 rpm. The supernatant was collected for fluorescence analysis. Then, the supernatant urine samples were spiked with standard Pb^2+^ and Epn solutions. Every sample was measured three times, and the results are listed in Table 3. Satisfactory recoveries of Pb^2+^ and Epn were obtained in the ranges of 97.65 to 99.26% and 96.70 to 97.21%, respectively. This shows that our fluorescent sensor could accurately detect Pb^2+^ and Epn concentrations in urine samples.

**Table 3:** The determination of Pb^2+^ ion and Epn drug concentrations in RCS using the proposed sensor

Sample	Spiked (nM)	Detected ± SD (nM)	Recovery (%)
**Pb** ^**2+**^			
Urine-1	2	1.953^*x*^ ± 0.43^*y*^	97.65
	4	3.955^*x*^ ± 0.51^*y*^	98.87
	6	5.956^*x*^ ± 1.29^*y*^	99.26

**Epn**			
Urine-2	6	5.802^*x*^ ± 0.63^*y*^	96.70
	8	7.752^*x*^ ± 0.56^*y*^	96.90
	10	9.721^*x*^ ± 1.43^*y*^	97.21

## 4. Conclusions

In summary, we have reported a novel Turn-ON fluorescent biosensor for the sequential detection of Pb^2+^ and the cancer drug Epn based on self-assembled NMc-CuTA/LFGr-ssDNA/AO. The proposed sensor utilizes the remarkable fluorescence quenching abilities of NMc-CuTA *via* interactions with an LFGr-ssDNA/AO probe, which has been extensively studied as a versatile sensing platform. The system works *via* a mechanism involving intermolecular structural changes in LFGr-ssDNA upon the introduction of Pb^2+^ and Epn, leading to the recovery of the fluorescence of AO. The changes in fluorescence intensity with specificity as a result of ssDNA were used for the selective detection of Pb^2+^ ions and Epn with detection limits of 1.5 and 5.6 nM, respectively, which are superior to the values for earlier reported sensing systems. Therefore, this sensing system holds great potential for the practical testing of complicated samples (like human urine) with satisfying results. We anticipate that this report of a novel fluorescent biosensor could prompt the development of more sensitive, simple, highly selective, and label-free methods for Pb^2+^ and Epn detection by the scientific community. Moreover, this sensor strategy can be extended to detect other heavy metal ions and other biomolecules *via* modifications to the specific oligonucleotide base sequence.

## Conflicts of interest

The authors declare no competing financial interests.

## Supplementary Material

RA-011-D1RA00939G-s001
