# Supplementary material for: A Turn-ON fluorometric biosensor based on ssDNA immobilized with a metal phenolic nanomaterial for the sequential detection of Pb(ii) and epirubicin cancer drug
Source: RSC Adv. 2021 Mar 29;11(20):12361–73. doi: 10.1039/d1ra00939g (PMC8696957; doi:10.1039/d1ra00939g)
Supplement: RA-011-D1RA00939G-s001 [file RA-011-D1RA00939G-s001.pdf]

Electronic Supplementary Material (ESI) for RSC Advances.
This journal is © The Royal Society of Chemistry 2021

Electronic Supplementary Information

Turn-ON Fluorometric Biosensor based on ssDNA immobilized with Metal Phenolic Nanomaterial for Subsequent Detection of Pb (II) and Epirubicin Cancer Drug

A. Arunjegan[a], P. Rajaji[a], S. Sivanesan[b], P. Panneerselvam[a*]

[a*] Department of Chemistry, SRM Institute of Science and Technology, Kattankulathur, Tamil Nadu, India-603 203.

[b] Department of Applied Science and Technology, A.C Technology, Anna University, Chennai, India 600 025

[*] Corresponding author & E-mail address:
panneerp1@srmist.edu.in,panneerchem82@gmail.com, Phone: +91 9688538842

List of Figures

Figure S1. BET analysis for NMc-CuTA

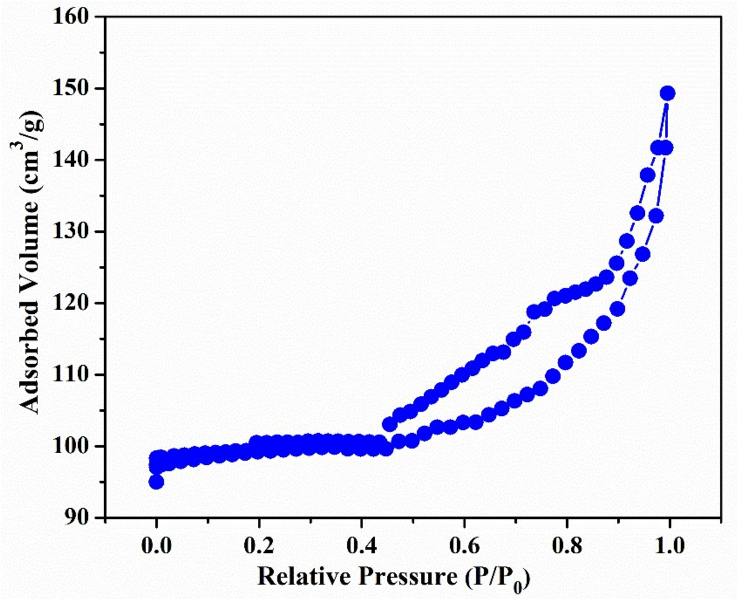

Figure S1. BET analysis for NMc-CuTA
